# Efficacy of the epidural blood patch for the treatment of post lumbar puncture headache BLOPP: A randomised, observer-blind, controlled clinical trial [ISRCTN 71598245]

**DOI:** 10.1186/1471-2377-5-12

**Published:** 2005-07-05

**Authors:** R Oedit, F van Kooten, SLM Bakker, DWJ Dippel

**Affiliations:** 1 Erasmus Medical Centre, Rotterdam, The Netherlands; 2 Amphia General Hospital, Breda/Oosterhout, The Netherlands

## Abstract

**Background:**

Post dural punction headache (PDPH) occurs in 10% to 40% of the patients who had a lumbar puncture. Its symptoms can be severe and incapacitating. The epidural blood patch is widely accepted as the treatment of choice for postdural puncture headache. Uncontrolled studies report rapid recovery after patching in 90% to 100% of treated patients. However, sufficient evidence from randomised, controlled clinical trials is lacking.

**Methods:**

BLOPP (blood patch for post dural puncture headache) is a randomised, single centre, observer-blind clinical trial. Patients with PDPH for at least 24 hours and at most 7 days after lumbar puncture will be randomised to treatment with an epidural blood patch (EDBP) or to conventional treatment, i.e. 24 hours bed rest and ample fluid intake. PDPH 24 hours after treatment, classified on a 4-point scale (no, mild, moderate, severe) is the primary outcome. The secondary outcome is the presence of PDPH 7 days after treatment. We estimated that a sample size of 2 × 20 patients would provide us with a power of 80% to detect a relative reduction in number of patients with persisting PDPH after 24 hours of 50% at the usual significance level α = 5%, taking into account that in approximately 10% of the patients the PDPH will have resolved spontaneously after one day.

**Discussion:**

The EDBP is accepted as the treatment of choice for PDPH although randomised, controlled data is scarce. Our randomised, observer-blind clinical trial enables us to compare the efficacy of two clinically practiced methods of PDPH treatment; EDBP versus conventional treatment, as they are applied in clinical practise.

## Background

Headache complicates approximately 10 to 40% of dural punctures [1]. This postdural puncture headache (PDPH) is typically orthostatic; provoked or aggravated by a vertical or upright position and relieved by a horizontal position. PDPH is probably caused by cerebral spinal fluid leakage through the dural rent, into the epidural space. The leakage causes a decrease in CSF pressure and volume, leading to traction on pain-sensitive structures in an upright position. Besides of headache, the patient may complain of diplopia, tinnitus, dizziness, and myalgia. PDPH may occur immediately after spinal tap, but it starts within 48 hours after the procedure in more than 90% of the patients. PDPH and accompanying symptoms are self-limiting. They generally resolve within 7 days or less, in 80% of the cases. In a small minority of cases, the symptoms may persist for weeks or even months [2]. During an episode of PDPH the patient may be completely incapacitated and confined to bed. Obviously this has financial, social and psychological repercussions.

Different prophylactic measures such as: small needle size, the use of Sprotte's needle, reinsertion of the stylet before withdrawing the needle, and direction of the brevel perpendicular to the dura, have all been shown to reduce the occurrence of PDPH [3-6]. If, despite the prophylactic measures, PDPH occurs, epidural blood patch (EDBP) may be a beneficial therapeutic intervention. EDBP has gained popularity as a therapeutic measure for PDPH. It involves the injection of 10–20 ml of autologous blood into the epidural space around the site of the spinal tap. Gormly introduced this technique in the 1960's [1]. He noticed that inadvertent bloody spinal taps were less often complicated by PDPH. He theorised that the epidural bleeding might lead to clot formation over the dural rent, preventing CSF leakage into the epidural space. He therefore continued to treat 6 subjects suffering from PDPH with EDBP, locating the epidural space with the hanging-drop or loss of resistance method. All 6 subjects were relieved of their complaints.

Many observational studies followed; they reported success rates of the EDBP for PDPH between 70% and 90% [7-12]. Seven controlled trials concerning prophylactic treatment have been published [13-19]. One of these studies was not blinded [18], three were not randomised [14-16], one was only reported as an abstract [17]. One other study [13] compared prophylactic EDBP versus no blood patch among obstetric patients and reported a high success rate. In this study adverse effects were not mentioned, which prevents firm conclusions. The latest study [19], in which prophylactic EDBP was compared to a sham procedure in a double blind setting, showed no decrease in the incidence of PDPH between the two groups. The effectiveness of prophylactic treatment does not seem to have been established firmly.

Only one randomised and blinded trial concerning the therapeutic effect of EDBP has been reported [20]. In this study 12 patients, suffering from PDPH for more than 4 days, despite conservative treatment following lumbar puncture, spinal anaesthesia or myelography, were randomly allocated to EDBP or sham treatment. In the placebo group none of the patients noted complete relief of pain. In the treatment group 5 of the 6 patients obtained immediate relief. Subsequently placebo group patients were also treated with an EDBP, resulting in complete relief of PDPH in all patients. The size of the study, the crossover effect, and the absence of any documentation regarding the effectiveness of blinding of the observers and patients, makes it difficult to draw firm conclusions from this study.

A Cochrane review on prophylactic and therapeutic blood patching [21] argued that further randomised trials of epidural blood patching must be carried out before the balance of risks and benefits of this intervention can be properly assessed".

Therefore, we decided to conduct a randomised, controlled, clinical trial comparing the efficacy of the EDBP with conservative treatment, consisting of 24 hours of bed rest and adequate fluid intake for the treatment of PDPH.

## Methods

### Inclusion criteria

To be included in the study, patients should have PDPH longer than 24 hours and not longer than 7 days after a diagnostic spinal tap. Furthermore patients should be aged 18 years or older. Written informed consent is required.

### Exclusion criteria

Excluded are patients with relative contra-indications for lumbar puncture: hemorrhagic diathesis and space-occupying intracranial lesions, and patients with a body temperature over 38° Celsius.

### Design

This is an observer-blind prospective, controlled and randomised parallel group study.

### Active treatment

Patients allocated to active treatment will receive an EDBP on the day of randomisation. The subject is placed in the lateral position, after which the back is flexed, sterilised and draped. Sterile gloves are used. A needle (Spinocan canule: 0.9 × 88 mm/206 × 3.5) is placed in the epidural space, using the loss of resistance technique. Subsequently, 20 cc of blood is than drawn from the antecubital vein, and injected slowly into the epidural space, after which the needle is removed. The subject is held in the supine position for a few minutes, after which there are no further restrictions.

### Control treatment

Conservative treatment consists of the advice to take 24 hours bed rest and drink at least 2.0 litres of fluid a day. The use of painkillers is not prohibited. Treatment with EDBP is not an option during the study period of 7 days, not even when conservative treatment fails.

### Outcomes

The primary outcome is whether or not headache is present at 24 hours after the start of treatment. Headache is classified on a 4-point scale (no, mild, moderate, severe). *Mild headache *is defined as: postural headache slightly restricting daily activities. The patient is not confined to bed and there are no associated symptoms. *Moderate headache: *postural headache confining the patient to bed for part of the day. Associated symptoms are not necessarily present. *Severe headache: *postural headache where the patient is bedridden for the entire day and associated symptoms are always present. The associated symptoms are: nausea, vomiting, dizziness, hearing loss, hyperacusis, tinitus, photophobia, diplopia, stiffness of the neck and scapular pain [2].

Secondary outcome measures are the presence of headache at day seven after the start of treatment, and the number of days until headache subsides.

### Treatment complications

A systematic assessment of complications of EDBP will be carried out. Back pain will be assessed systematically, and classified as no, mild moderate, severe.

### Statistical analysis

The number of patients with headache at 24 hours after start of treatment will be compared between the two treatment strategies. The effect of treatment on the occurrence of the headache will be expressed as an odds ratio with 95% confidence interval. Adjustment for the effect of potential confounders, such as age and sex, will be made by multiple logistic regression analysis. The number of days until headache relief will be compared with Kaplan-Meier survival analysis techniques; observations will be censored at the end of the study period, i.e. 7 days. Adjustments for the effect of potential confounders will be made with proportional hazards regression [22].

### Sample size

A trial with 20 patients in each treatment group will provide us with a power (1-β) of 80% to detect a relative reduction in number of patients with persisting PDPH after 24 hours of 50% (OR = 12) and a power of 99% to detect a relative risk reduction of 80%, (OR = 81, at the usual significance level α = 5%, taking into account that in approximately 10% of the patients the PDPH will have resolved spontaneously after one day.

### Recruitment of eligible patients

Patients receiving a diagnostic dural puncture are informed about the possibility of developing PDPH. They all receive written information about the study. A subject suffering from headache after a dural puncture will be advised to contact his physician. The physician evaluates whether the headache is a PDPH or not. When the conclusion is that the subject is suffering from PDPH, he or she is asked to participate. Randomisation is done by a telephone call, by the investigator.

### Randomisation procedure

The randomisation procedure is carried out by one of the investigators (RO or FvK). During the telephone contact, while the patient is entered into the computer database the treatment allocation (based on a random number generator) is provided by the computer. The patient cannot be entered twice into the study, nor can the entry be erased.

### Ethical considerations and informed consent

The local medical ethics committee and review board approved this study. Written informed consent will be obtained from all patients, by asking them to return the form by mail or in person, after the randomisation by telephone.

### Baseline data

At baseline, patient demographics age and gender will be registered. Both have been proven to be independent risk factors for the development of PDPH. Clinical characteristics, such as headache characteristics, other complaints, and use of analgesics will be noted. Information concerning needle type, size and re-insertment of the stylet before withdrawing is registered, since all three factors are independent risk factors for the development of PDPH [3-5]. Furthermore the severity of the PDPH is registered on a four-point scale.

### Follow-up and blinding

The follow-up visits are carried out by telephone, by a research nurse at the trial office, 24 hours after randomisation, and at 1 week after randomisation. The research nurse is kept blind to the treatment allocation. At the beginning of the telephone interview the patients are instructed not to inform the observer of the treatment they received. The effectiveness of the blinding will be checked in a sample of 12 patients, by letting the research nurse fill in a forced choice item indicating the treatment allocation.

## Discussion

The EDBP, is widely accepted as the treatment of choice for PDPH. In a review of the literature on this subject however, we found only little evidence to justify its use in general practice [21]. Therefore, we decided to conduct an observer blinded, randomised, controlled, study comparing the therapeutic efficacy of the EDBP to the efficacy of conservative treatment.

Our study design not only enables us measure the effectiveness of the EDBP, but also its efficiency. The number of days spent with headache after treatment in the two groups will be characterised as incapacitated-days. Immediate relief of headache, and reduction of the number of incapacitated-days is the ultimate goal of this treatment. By taking into account the adverse effects of EBPD, most notably lower back pain, we will be able to make a more realistic estimate of the overall effect of EPBD.

The observer-blind method we have chosen enables us to compare the two treatment strategies of interest as if they are carried out in normal practise, without the artificial circumstances created by sham treatment, in futile attempts to maintain patient blinding. So a genuine comparison is made between the experimental treatment and normal clinical practise. Because patients are of course aware of the nature of the treatment, the study design limits our ability to assess the placebo effect of the epidural blood patch. One may argue that this may lead to overestimation of the effect of the blood patch. We think that for a treatment of a self/limiting condition this is not really a drawback. Another potential limitation is the small sample size of our study. Although our trial will turn out to be the largest randomised study of therapeutic EDBP ever, a sample size of 20 patients in each treatment group will provide us sufficient power (1-β = 80%) to detect a relative reduction in the risk of PDPH after 24 hours of 50%. Much depends therefore on our assumption that only in approximately 10% of the patients the PDPH will have resolved spontaneously after one day. Currently, the study is well underway, and we expect to be able to report its results in the end of 2005.

## Conclusion

EDBP is accepted as the treatment of choice for PDPH although randomised, controlled data is scarce. Our randomised, observer-blind clinical trial enables us to compare the efficacy of two clinically practiced methods of PDPH treatment; EDBP versus conventional treatment, as they are applied in clinical practise.

## List of abbreviations

CSF: Cerebrospinal fluid

EDBP: Epidural blood patch

PDPH: Post dural punction headache

## Competing interests

The author(s) declare that they have no competing interests.

## Authors' contributions

RO participated in the study design and acquisition of data, and drafted the manuscript. FK participated in data acquisition, coordination of the study, and helped to draft of the manuscript. SB participated in the conception of the study, and in data acquisition. DD participated in the conception of the study, data acquisition, and drafting of the manuscript. All authors have seen and approved the final manuscript.

**Figure 1:**
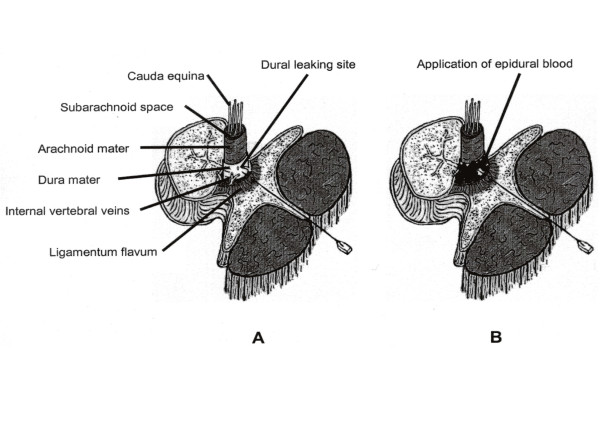
Schematic section through the vertebral column, showing the cauda equina and its covering membranes with the dural leakage site before (A) and after (B) application of the epidural blood patch.

**Table 1:** Therapeutic studies of EDBP for PDPH

**Author**	**Number of patients**	**Type of study**	**Blinding**	**Time window to inclusion**	**Follow-up**	**Outcome**	**Adverse effects**
Safa-Tisseront *2001 [8]*	527 (504 analyzed)	Therapeutic, prospective, non-randomised, observational	NA	4 days (1–53)	15 days	75% complete relief, 18% incomplete relief, 7% failure	Fever in 3 patients
Williams *1999 [12]*	55 (7 prophylactic, 41 therapeutic EDBP, 7 conservative treatment)	Retrospective, non-randomised, observational	NA	<1 week	?	34% complete relief, 54% incomplete relief, 12% failure	Back pain in 3 patients
Banks *2001 [9]*	100 (81 pdph7 prophylactic, 58 therapeutic EDBP, 23 conservative treatment)	Prospective, non randomised, observational	NA	0–3 days	?	67% complete relief, 28% incomplete relief, 5% failure	22% back pain
Taivainen *1993 [7]*	81 (55 patients 10 ml, 26 patients 10–15 ml EDBP)	Prospective, partly randomised, observational	NA	0–10 days	1 week	Initial relief 91%, permanent relief 61%	25% back pain
Vercauteren *1999 [11]*	190 (186 EDBP, 4 atypical symptoms)	Retrospective, non randomised	NA	>24 h after symptoms	1 day	Initial relief 99%, permanent relief 73%	?
Stride *1993 [10]*	34819 (461 PDPH, 137 EDBP attempted, 135 completed)	Retrospective, non randomised	NA	2 days	?	Initial relief 90%, permanent Relief 64%	?
Seebacher *1989 [20]*	12 (6 EDBP, 6 sham treatment)	Prospective, randomised	Yes	>4 days	1 day	EDBP 83% relief sham treatment 0% relief	Back pain

## Pre-publication history

The pre-publication history for this paper can be accessed here:


